# Time to start a research agenda on electoral integrity within political organizations

**DOI:** 10.12688/openreseurope.23702.1

**Published:** 2026-05-02

**Authors:** F. Ramón Villaplana, Gabriela Borz, Jasmin Fitzpatrick, Marco Lisi, Oscar Barberà

**Affiliations:** 1 Political Science, Social Anthropology and Public Finance, University of Murcia, Murcia, Region of Murcia, 30008, Spain; 2 Government & Public Policy, University of Strathclyde, Glasgow, Scotland, G1 1XQ, UK; 3 Babeș-Bolyai University, Cluj-Napoca, Romania; 4 German Politics and Political Sociology, Johannes Gutenberg University Mainz Department of Political Science, Mainz, Rhineland-Palatinate, 55128, Germany; 5 Political Studies, Nova University of Lisbon Faculty of Social Sciences and Humanities, Lisbon, Lisbon, 1069-061, Portugal; 6 Constitutional Law and Political Science, Universitat de Valencia, Valencia, Valencian Community, 46010, Spain

**Keywords:** electoral integrity, political organizations, civil society, e-voting, digital politics.

## Abstract

Electoral integrity scholarship has made noteworthy progress studying national elections, yet a vital blind spot persists: the governance decisions conducted by political organizations –such as political parties, trade unions, civil society organizations and youth organizations– remain almost entirely beyond its analytical scope. These political actors collectively constitute a vital intermediate layer of democratic life, shaping who leads, whose voices are amplified, and how citizens relate to the political system. This open letter warns that their internal electoral processes are a site of frequent malpractice, that existing regulatory frameworks are wholly inadequate, and that the rapid digitalization of processes has introduced new vulnerabilities requiring urgent attention. We call on researchers, funding bodies, and policymakers to develop a new, interdisciplinary research agenda on electoral integrity within and across all major forms of democratic organization.

## Plain Language summary

Every year, millions of people head to the polls to choose their representatives. Scholars, observers, and journalists scrutinize every step of the process: counting, campaigning, rules, etc. But long before any of that happens, quieter elections take place in the meeting rooms and online platforms of political parties, trade unions, civic and youth organizations. These are the moments when leaders are chosen, candidates are selected and the direction of political life is set.

This is the blind spot that we are now calling out. Political organizations form the backbone of democratic life. They are the bridge between citizens and the state, the places where political careers begin and collective voices take shape. Yet when these organizations hold their own internal elections, the standards we demand of national votes simply do not apply. There are few rules, little oversight and almost no research. The consequences are real: internal elections within parties and organizations are, by many accounts, frequently manipulated. Also, as organizations rush to digitalize their internal processes, moving votes and decisions online, fresh vulnerabilities are opening up faster than anyone can address them. The risks of hacking, fraud and digital exclusion are growing.

We are issuing a challenge: to scholars, to practitioners, to funding bodies and to governments. It is time to treat the internal life of political organizations as a serious subject of study and policy, because democracy is not just what happens on election day. It is built, shaped, and sometimes quietly broken in the everyday decisions of the organizations that sit between citizens and power. Right now, that process is largely in the shadows. It doesn’t have to be.

## 1. Electoral integrity within organizations: a concerning gap in literature and practice

Electoral integrity scholarship has established itself as one of the most productive growth areas in electoral studies over the past two decades. Foundational works by Birch (2011) and Norris (2014, 2015) provided the conceptual architecture –distinguishing electoral malpractice from maladministration, mapping manipulation across the full electoral cycle, and demonstrating the far-reaching consequences of integrity failures for democratic legitimacy and citizen trust (see Windecker 2025 for a comprehensive overview). The Electoral Integrity Project generated the most systematic cross-national data to date ( Garnett and James, 2025) and represents the field’s maturation. All of this work, however, shares a defining constraint: it studies elections administered by states, at national or sub-national level. We argue that integrity as a principle can be defining for the political behaviour of individuals, leaders and management structures across most types of institutions.

The integrity principle is pro-active, highly relevant in anti-corruption policies and forms the rational basis of public trust ( Kirby, Robinson and Cadzow, 2018). As the integrity of national electoral processes impacts on legitimacy, trust, confidence and satisfaction with democracy ( Norris, 2015), we expect the integrity of political organizations to have the same impact at the individual level: to increase trust in politicians and ultimately to contribute to democratic quality. Organizational integrity lies between institutional integrity (system level) and electoral integrity (process at the national level), and focuses on aligning political actors’ values and rules with actions. It ensures that organizations behave ethically, reliably and transparently ( Huberts, 2018). Therefore, we aim to expand this framework at the meso-level of politics.

Between the citizen and the state sits a dense intermediate layer of organizations that are themselves internally democratic –or at least claim to be. Political parties are the central transmission belts of representative democracy, holding primaries and leadership contests that directly determine who occupies public office ( Katz and Mair, 1995; Hazan and Rahat, 2010). Trade unions elect officers and executive bodies who negotiate on behalf of millions of workers and exercise substantial public power ( Baccaro, Benassi and Meardi, 2019). Non-governmental organizations and civil society bodies elect governing boards, approve strategic directions, and make decisions whose democratic legitimacy is increasingly contested ( Kalm, Strömbom and Uhlin, 2019). And youth organizations affiliated with parties, student unions, and independent youth movements elect leaders and adopt political positions that shape the next generation of public representatives. The integrity of electoral and managerial processes within all of these organizations is a matter of public consequence –yet it has attracted almost no systematic attention.

The intra-party democracy literature has examined party candidate selection and leadership contests with growing sophistication ( Kenig, 2009; Cross and Pilet, 2015; Sandri and Seddone, 2021), but has focused almost exclusively on questions of design –who participates as a selectorate, what procedures exist– while largely bracketing questions of procedural integrity: whether these processes are conducted fairly, impartially, and according to transparent and verifiable rules. Since Lipset, Trow and Coleman’s (1956) seminal study of the International Typographical Union and Edelstein and Warner’s (1975) comparative analysis, the literature on trade unions has mostly not been brought into conversation with electoral integrity frameworks. The governance of NGOs and youth organizations has been examined from the perspectives of accountability and participation ( Fung, 2003; Ebrahim, 2005), but never through the lens of electoral integrity in their internal processes. These fields have developed in parallel, and their convergence is long overdue.

The organizational scope of the research gap is significant. In advanced democracies, major trade unions represent an important –but diverse– percent of the workforce ( OECD, 2025). Their internal elections can mobilize memberships in the hundreds of thousands. Party youth organizations serve as nurseries of political elites and socialization mechanisms for future participation ( Hooghe et al., 2004; Elliott and Earl, 2018). Large NGOs and advocacy organizations operate across borders, manage substantial resources, and exercise growing political influence –yet their governance elections are subject to no external oversight whatsoever. The democratic quality of these processes matters for the political system as a whole, not just for the organizations themselves.

## 2. Malpractices across organizations and the need for regulation and observation

Far from being exceptional, irregularities in internal organizational elections are a recurrent if under-documented phenomenon across all the organizational types under consideration. The vocabulary developed to study electoral malpractice at the state level –including the manipulation of eligibility lists, abuse of incumbency resources, opaque counting, undue pressure on voters, and outright falsification of results ( Birch, 2011; Lehoucq, 2003)– applies with equal force to internal organizational elections, with adaptations required for each organizational context.

Reported irregularities in party primaries, party referendums and leadership contests are as old as contemporary political actors themselves ( Duverger, 1954). This includes the manipulation of membership rolls to admit or exclude eligible voters, the mobilization of incumbency resources –party staff, databases, and infrastructure– to advantage one faction, pressure on delegates and local officers, the absence of independent auditing of results, etc. Legal frameworks are almost universally inadequate: most democracies grant parties wide organizational autonomy under freedom of association doctrine. Despite receiving substantial amounts of public funding in most countries, there often are only minimal statutory requirements for internal governance ( Biezen I van and Piccio, 2013). When a member challenges the outcome of an internal ballot, recourse is typically limited to internal bodies that are rarely independent of the competing factions.

The history of union democracy is also inseparable from the history of electoral manipulation. Michels (1911) documented systematic oligarchical tendencies in socialist parties and unions alike. Subsequent empirical work in comparative union democracy found that incumbent officers routinely exploited control over internal communications, financial resources, and administrative machinery to entrench themselves ( Lipset et al., 1956; Edelstein and Warner, 1975). Contemporary cases in multiple countries document ballot irregularities, the exclusion of rival slates on procedural pretexts, and the use of employer-facing relationships to discipline dissenting member voters. Regulatory regimes vary widely: some legal systems (notably the United States under the Labor-Management Reporting and Disclosure Act, and the United Kingdom under successive trade union legislation) provide for external oversight and certification of union elections, but most others impose no such controls ( Baccaro, Benassi and Meardi, 2019). The comparative landscape of union election regulation has never been systematically mapped.

Furthermore, non-governmental organizations present a structurally different challenge. Unlike parties and unions, whose democratic internal governance is at least nominally required by their claim to represent a constituency, NGOs often claim democratic legitimacy on the basis of their causes rather than their governance. Yet large NGOs and advocacy organizations exercise significant public influence –lobbying governments, shaping policy agendas, managing major resources– and their boards and spokespersons are typically elected by restricted selectorates of members, donors or even affiliate organizations ( Ebrahim, 2005). Leach (2005) generalized Michels’ iron law of oligarchy based on legitimate and illegitimate forms of formal and informal power present in “collectivist-democratic organizations”. The integrity of elections in these organizations is subject to no external scrutiny, and there is no established normative framework against which they could be assessed. The same applies to international federations of civil society organizations, which elect steering bodies whose legitimacy rests entirely on unverified internal processes.

Political youth organizations –party youth wings, student unions, and autonomous youth movements– constitute a particular organizational type. Their memberships are large, transient and heterogeneous. Their elections are often the first experience of organizational democracy for participants and their outcomes shape the pipeline of political elite recruitment ( Hooghe et al., 2004). Yet they operate with minimal institutional supervision and high susceptibility to manipulation by parent organizations or factional interests within the broader party or senior organization. Student unions, which typically hold elections with high symbolic salience and media visibility, also have documented histories of electoral irregularities ranging from vote-buying and intimidation to fraudulent candidate registration. These cases receive episodic journalistic attention but no systematic scientific analysis (but see Gordon and Taft, 2011).

Across all these organizational types, a common mechanism connects integrity failures to broader democratic consequences: when internal processes are perceived as flawed, they erode the trust that members –and the wider public– place in the organization as a democratic actor ( Norris et al., 2016; Carreras and Irepoglu, 2013). A party whose candidate emerged from a rigged primary, a union whose leader was never fairly elected, a youth organization whose head was installed by a parent party, an NGO whose board perpetuates itself without genuine competition … Each of these cases represents a delegitimization of the organization concerned and, in aggregate, a corrosion of the democratic fabric within which these organizations operate.

## 3. The digital dimension: shared vulnerabilities and organization-specific risks

The digitalization of internal organizational elections has accelerated markedly since the 2010s, driven by the diffusion of online voting platforms, messaging applications, social media, and dedicated member-management software. Political parties have been among the most visible adopters, with cases such as Podemos in Spain, the Italian Five Stars Movement and some Pirate parties attracting academic and public attention as experiments in digital intra-party democracy ( Gerbaudo, 2019; Barberà et al., 2021; García-Lupato and Meloni, 2023). But the digitalization of internal elections extends well beyond parties: major trade unions in several countries now use digital platforms to ballot their memberships; student unions have pioneered online voting in academic settings; and NGOs increasingly use digital governance tools for board elections and member consultations and to achieve their political goals ( Fitzpatrick, 2018).

Party actors are well aware of the cybersecurity vulnerabilities introduced by digital internal elections but lack the institutional capacity to address them ( Paulis et al., 2025). There is every reason to expect that this dilemma is at least as acute –and probably more so– for trade unions, youth organizations, and NGOs, which typically have fewer technological resources, less specialized staff and even weaker oversight standards than parties. The integrity challenges introduced by digitalization are therefore not a party-specific problem but a systemic challenge for the entire intermediate organizational layer of democracy, alongside democracy at macro level ( Garnett and James, 2020; Dueñas-Cid et al., 2026).

These challenges take both general and organization-specific forms ( Nostitz, F von, Sandri and Barrat, 2021). In general, digital voting systems may lack the transparency and auditability of in-person balloting: membership databases can be manipulated without a paper trail, proprietary platforms impede independent verification and social media enables coordinated disinformation and voter intimidation ( Chadwick and Stromer-Galley, 2016; Vaccari and Chadwick, 2020). More specifically, party primaries and referendums face specific risks of algorithmic manipulation of voter rolls and targeted micro-mobilization by factional actors; trade union ballots face risks of employer interference via digital platforms; youth organizations face vulnerabilities around identity verification in high-turnover memberships; and NGO board elections face risks of unauthorized access to restricted voting systems by competing donor or affiliate groups.

The emergence of generative artificial intelligence adds a further and yet barely studied layer of risk (but see Novelli et al., 2024; Borz et al., 2025; Yardimci-Geyikci et al., 2026). AI tools could be used to generate synthetic testimonials from fabricated members, automate coordinated disinformation campaigns targeted at specific organizational constituencies, manipulate internal deliberation processes that precede formal votes, or mimic communications from organizational leadership to suppress turnout among targeted groups ( Altman, 2026). None of the existing normative or regulatory frameworks for electoral integrity –which are themselves only beginning to grapple with AI in the context of general elections– address these risks in the internal organizational setting.

## 4. A call for action: building a new research agenda

We call on the political science community, legal academics, computer scientists, civil society researchers and research funding bodies to recognize the integrity of internal elections across political and civil society organizations as a shared and compelling priority. The research agenda we are proposing is necessarily interdisciplinary –it cannot be advanced from within any single disciplinary tradition– and must be developed in direct engagement with practitioners. We propose that it be organized around the following axes:

• Definition, operationalization and cross-organizational comparison. The field needs a unified yet organizationally sensitive conceptual framework. Core concepts from electoral integrity literature –malpractice, manipulation, observer independence, verifiability, legitimacy– must be adapted to the specific features of different organizational types: their membership structures, legal statuses, accountability relationships and roles in the democratic system. Crucially, this framework must be comparative across organizational types, enabling researchers to identify shared patterns and organization-specific variations. The long-standing separation between party politics and civil society studies has prevented precisely this kind of cross-organizational and interdisciplinary perspective.
• Normative benchmarking. Similar to the international standards developed for general elections, there is a need for internationally recognized normative benchmarks defining what integrity in internal organizational elections should look like. It is important to look at this process in a systematic way, not only across distinct stages –elaboration of rules, campaign, voting and acceptance– but also regarding distinct principles such as contestation, deliberation, participation and adjudication. Such standards would serve as reference points for self-regulation by organizations, external assessment by civil society and researchers, and potentially legislative reform. Developing them requires engaging the relevant international bodies and major civil society networks, in addition to academic communities.
• Legal and self-regulatory analysis. A comparative mapping of the legal frameworks governing internal elections across organizational types and jurisdictions is urgently needed. For parties, this means building on the emerging party rules literature ( Biezen I van and Piccio, 2013; Poguntke et al., 2016; Gauja, 2017; Scarrow and Casal Bértoa, 2026) to assess which regulatory models best balance organizational autonomy with democratic accountability. For unions, it means systematically comparing the divergent regulatory regimes that exist across democratic systems. For NGOs and youth organizations, it means confronting an almost complete regulatory vacuum and developing principled arguments for what appropriate oversight might look like. The goal is not uniformity but the identification of good practices and the development of principled criteria for reform.
• Digital integrity standards. Developing technical and procedural standards for verifiable digital internal elections is an interdisciplinary challenge requiring collaboration between political scientists, computer scientists, cybersecurity specialists and lawyers. Existing standards for e-voting in public elections provide a starting point but must be rethought for the organizational context: lower resource environments, higher membership turnover, greater difficulty of independent certification and the specific manipulation incentives present in partisan and factional organizational settings. AI risks to internal organizational democracy deserve particular attention, as this is a frontier that has not been addressed in any existing normative framework (but see Novelli et al., 2024; Borz et al., 2025; Yardimci-Geyikci et al., 2026).
• Pilot studies and comparative empirical mapping. Systematic cross-national data on the prevalence, typology and consequences of electoral malpractice in internal organizational elections do not currently exist for any of the organizational types under consideration. Building such data requires surveys of organization members and officials, analysis of statutes and judicial records, expert assessments, and direct observation of internal processes. Methodologically, researchers will need to navigate significant access challenges: organizations have strong incentives to manage information about internal irregularities. Triangulating across multiple data sources –including litigation records, whistleblower accounts and comparative statutory analysis– will be essential. Furthermore, the lack of public opinion data –i.e., what citizens think of the quality of these electoral processes, what is the level of confidence across distinct population groups– requests additional attention for achieving a full picture of the phenomenon.
• Researcher-practitioner engagement. Research that is developed without engagement with the practitioners who administer internal elections –party officials, union returning officers, NGO secretariats, youth organization boards– will fail to understand the real constraints and incentives that shape electoral processes and their idiosyncrasies. Equally, think tanks that monitor democratic quality, journalists who investigate organizational governance and practitioners who manage internal election disputes are essential partners. The research agenda we are proposing must be co-developed with these actors, not merely communicated to them after the fact.

Political parties, trade unions, civil society organizations and youth organizations are not peripheral to democracy: they are its connective tissue. They are where many citizens first experience democratic life: where political identities are formed, where leaders are recruited and tested and where collective interests are aggregated into political demands. The internal democratic quality of these organizations is therefore not a matter of private organizational governance; it is a constitutive dimension of democracy itself. Michels’s (1911) iron law of oligarchy identified the structural pressure toward elite entrenchment in all democratic organizations. More than a century later, we have still not developed the necessary knowledge to understand when and how electoral malpractices within these organizations occur, what conditions enable or prevent them or what normative principles would most effectively constrain them.

The democratic legitimacy of parties has been under sustained public scrutiny ( van Biezen et al., 2012; Ignazi, 2014; Borz and Janda, 2020), the representativeness of unions is widely contested, youth organizations are frequently dismissed as appendages of parent parties and the accountability of large NGOs is regularly challenged. One underappreciated driver of all these crises of legitimacy may be the perception –often well-founded but rarely documented– that internal elections are conducted unfairly. Addressing this perception requires, first and foremost, knowing whether it is accurate: systematic empirical research on the integrity of internal organizational elections is the necessary foundation for any serious response.

We must recognize that the disconnect between democratic institutions and citizens runs through the intermediate organizations that are supposed to tie them. Therefore, we invite colleagues across political science, law, digital studies, and civil society research to join us in building the research agenda that this challenge demands.

## Ethics and consent

Ethical approval and consent were not required.

## Disclaimer

The views expressed in this article are those of the authors. Publication in Open Research Europe does not imply endorsement of the European Commission.

## Declaration of AI use

The corresponding author (FRV) used Claude Sonnet 4.6 (Anthropic) to assist with English grammar and style revision before submission.

## Data Availability

No data are associated with this article.

## References

[ref1] Altman D : The AI Democracy Dilemma. *Journal of Democracy.* 2026;37(1):32–44. 10.1353/jod.2026.a977942

[ref2] Baccaro L Benassi C Meardi G : Theoretical and empirical links between trade unions and democracy. *Economic and Industrial Democracy.* 2019;40(1):3–19. 10.1177/0143831X18781714

[ref3] Barberà O Sandri G Correa P , editors. *Digital Parties.* *The Challenges of Online Organisation and Participation.* Cham: Springer;2021. 10.1007/978-3-030-78668-7

[ref46] Biezen I van Piccio DR : Shaping intra-party democracy: On the legal regulation of internal party organizations. Cross WP Katz RS , editors. *The Challenges of Intra-Party Democracy.* Oxford: Oxford University Press;2013; pp.27–48. 10.1093/acprof:oso/9780199661879.003.0003

[ref47] Biezen I van Mair P Poguntke T : Going, going… gone? The decline of party membership in contemporary Europe. *European Journal of Political Research.* 2012;51(1):24–56. 10.1111/j.1475-6765.2011.01995.x

[ref4] Birch S : *Electoral Malpractice.* Oxford: Oxford University Press;2011. 10.1093/acprof:oso/9780199606160.001.0001

[ref5] Borz G Janda K : Contemporary trends in party organization: Revisiting intra-party democracy. *Party Politics.* 2020;26(1):3–8. 10.1177/1354068818754605

[ref6] Borz G Mitrea C Longhini A : Digital technology and electoral democracy: introducing the DIGIEFFECT project. *Open Access Government.* 2025;46:290–291. 10.56367/OAG-046-11926

[ref7] Carreras M Irepoglu Y : Trust in elections, vote buying, and turnout in Latin America. *Electoral Studies.* 2013;32(4):609–619. 10.1016/j.electstud.2013.07.012

[ref8] Chadwick A Stromer-Galley J : Digital media, power, and democracy in parties and election campaigns. *The International Journal of Press/Politics.* 2016;21(3):283–293. 10.1177/1940161216646731

[ref9] Cross WP Pilet J-B : *The Politics of Party Leadership: A Cross-National Perspective.* Oxford: Oxford University Press;2015. 10.1093/acprof:oso/9780198748984.001.0001

[ref10] Dueñas-Cid D Spycher-Krivonosova I Krimmer R : From Postal Ballots to E-Voting: Renewing the Definition and Typology of Convenience Voting. *Election Law Journal: Rules, Politics, and Policy.* 2026. 10.1177/15331296251411801

[ref11] Duverger M : *Political Parties. Their Organization and Activity in the Modern State.* New York: John Wiley and Sons;1954. Reference Source

[ref12] Ebrahim A : Accountability myopia: Losing sight of organizational learning. *Nonprofit and Voluntary Sector Quarterly.* 2005;34(1):56–87. 10.1177/0899764004269430

[ref13] Edelstein JD Warner M : *Comparative Union Democracy: Organisation and Opposition in British and American Unions.* London: George Allen and Unwin;1975. Reference Source

[ref14] Elliott T Earl J : Organizing the next generation: Youth engagement with activism inside and outside of organizations. *Social Media+ Society.* 2018;4(1). 10.1177/2056305117750722

[ref15] Fitzpatrick J : *Digital Civil Society. Wie zivilgesellschaftliche Organisationen im Web 2.0 politische Ziele verfolgen.* Berlin: Springer;2018. 10.1007/978-3-658-21433-3

[ref16] Fung A : Associations and democracy: Between theories, hopes, and realities. *Annual Review of Sociology.* 2003;29:515–539. 10.1146/annurev.soc.29.010202.100134

[ref17] García-Lupato F Meloni M : Digital intra-party democracy: An exploratory analysis of Podemos and the Labour Party. *Parliamentary Affairs.* 2023;76(1):22–42. 10.1093/pa/gsab015

[ref18] Garnett HA James TS : *The Oxford Handbook of Electoral Integrity (online edition).* Oxford: Oxford University Press;2025. 10.1093/oxfordhb/9780197777497.001.0001

[ref19] Garnett HA James TS : Cyber elections in the digital age: threats and opportunities of technology for electoral integrity. *Election Law Journal: Rules, Politics, and Policy.* 2020;19(2):111–126. 10.1089/elj.2020.0633

[ref20] Gauja A : *Party Reform: The Causes, Challenges and Consequences of Organizational Change.* Oxford: Oxford University Press;2017. 10.1093/acprof:oso/9780198717164.001.0001

[ref21] Gerbaudo P : *The Digital Party: Political Organisation and Online Democracy.* London: Pluto Press;2019. Reference Source

[ref22] Gordon HR Taft JK : Rethinking youth political socialization: Teenage activists talk back. *Youth & Society.* 2011;43(4):1499–1527. 10.1177/0044118X10386087

[ref23] Hazan RY Rahat G : *Democracy within Parties: Candidate Selection Methods and Their Political Consequences.* Oxford: Oxford University Press;2010. 10.1093/acprof:oso/9780199572540.001.0001

[ref24] Hooghe M Stolle D Stouthuysen P : Head start in politics: The recruitment function of youth organizations of political parties in Belgium (Flanders). *Party Politics.* 2004;10(2):193–212. 10.1177/1354068804040503

[ref25] Huberts LWJC : Integrity: What it is and Why it is Important. *Public Integrity.* 2018;20(sup1):S18–S32. 10.1080/10999922.2018.1477404

[ref26] Ignazi P : Power and the (il) legitimacy of political parties: An unavoidable paradox of contemporary democracy?. *Party Politics.* 2014;20(2):160–169. 10.1177/1354068813519350

[ref27] Kalm S Strömbom L Uhlin A : Civil Society Democratising Global Governance? Potentials and Limitations of “Counter-Democracy.”. *Global Society.* 2019;33(4):499–519. 10.1080/13600826.2019.1640189

[ref28] Katz RS Mair P : Changing models of party organization and party democracy: The emergence of the cartel party. *Party Politics.* 1995;1(1):5–28. 10.1177/1354068895001001001

[ref29] Kenig O : Democratization of party leadership selection: Do wider selectorates produce more competitive contests?. *Electoral Studies.* 2009;28(2):240–247. 10.1016/j.electstud.2008.11.001

[ref30] Kirby N Robinson T Cadzow L : *Investigating integrity: A multi-disciplinary literature review.* University of Oxford, Blavatnik School of Government;2018. Working Paper. Reference Source

[ref31] Leach DK : The Iron Law of What again? Conceptualizing Oligarchy across Organizational Forms. *Sociological Theory.* 2005;23(3):312–337. Reference Source

[ref32] Lehoucq F : Electoral fraud: Causes, types, and consequences. *Annual Review of Political Science.* 2003;6:233–256. 10.1146/annurev.polisci.6.121901.085655

[ref33] Lipset SM Trow M Coleman JS : *Union Democracy: The Internal Politics of the International Typographical Union.* Glencoe, IL: Free Press;1956. Reference Source

[ref34] Michels R : *Political Parties: A Sociological Study of the Oligarchical Tendencies of Modern Democracy.* New York: Hearst's International Library Co.;1911 [1915]. Reference Source

[ref35] Norris P : *Why Electoral Integrity Matters.* Cambridge: Cambridge University Press;2014. 10.1017/CBO9781107280861

[ref36] Norris P : *Why Elections Fail.* Cambridge: Cambridge University Press;2015. 10.1017/CBO9781107280908

[ref37] Norris P Frank RW Martínez i Coma F : *Contentious Elections: From Ballots to Barricades.* New York: Routledge;2016. 10.4324/9781315723068

[ref44] Nostitz F von Sandri G Barrat J : Regulating i-Voting Within Countries and Parties. Barberà O Sandri G Correa P , editors. *Digital Parties. Studies in Digital Politics and Governance.* Cham: Springer;2021. 10.1007/978-3-030-78668-7_3

[ref38] Novelli C Formisano G Juneja P : Artificial Intelligence for the Internal Democracy of Political Parties. *Minds & Machines.* 2024;34(36). 10.1007/s11023-024-09693-x

[ref39] OECD: *Membership of unions and employers' organisations, and bargaining coverage. Standing, but losing ground.* Paris: OECD;2025. 10.1787/fe47107c-en

[ref40] Paulis E Kins L Barberà O : The flip side of digital technologies in internal party elections: A cybersecurity dilemma?. *Innovation: The European Journal of Social Science Research.* 2025;38(3):1386–1411. 10.1080/13511610.2025.2461996

[ref41] Poguntke T Scarrow SE Webb PD : Party rules, party resources and the politics of parliamentary democracies: How parties organize in the 21st century. *Party Politics.* 2016;22(6):661–678. 10.1177/1354068816662493

[ref42] Sandri G Seddone A : *New Paths for Selecting Political Elites: Investigating the Impact of Inclusive Candidate and Party Leader Selection Methods.* London: Routledge;2021. 10.4324/9781003022893 Reference Source

[ref43] Scarrow SE Casal Bértoa F : Party research and party support aid: Conflicting visions of how parties function?. *Party Politics.* 2026. 10.1177/13540688261430702

[ref45] Vaccari C Chadwick A : Deepfakes and disinformation: Exploring the impact of synthetic political video on deception, uncertainty, and trust in news. *Social Media + Society.* 2020;6(1). 10.1177/2056305120903408

[ref48] Windecker P : *Perceptions of Electoral Integrity Back on the Drawing Board.* Wiesbaden: Springer;2025. 10.1007/978-3-658-49289-2

[ref49] Yardimci-Geyikci S Correa P Sandri G : AI and the Party: The Three Faces of Party Organization and Artificial Intelligence. *SSRN.* 2026. 10.2139/ssrn.5899882

