# Supplementary material for: Time to start a research agenda on electoral integrity within political organizations
Source: Open Res Eur. 2026 May 2;6:142. [Version 1] doi: 10.12688/openreseurope.23702.1 (PMC13231258; doi:10.12688/openreseurope.23702.1)
Supplement: Peer review history

# Reviewer report: Reviewer response for version 1

**DOI:** 10.21956/openreseurope.25660.r73645

**Published:** 2026-06-13

**Authors:** David Duenas-Cid

**Affiliations:** 1 Kozminski University, Warsaw, Masovian Voivodeship, Poland

Thank you for giving me the chance to review this text. The authors did a good job in synthesising many different ideas within a single piece of work, and turned it into a propositive (and necessary) research agenda. While acknowledging their work, I will bring a couple of criticisms. My aim with them is not to undermine the good work of the authors, but to highlight elements that (IMHO) could be improved to provide a better understanding of the topic. 

 My general impression is that this work opens an exciting and promising research avenue focusing on the internal democratic processes of political structures (political parties and organisations) that are meant to lead the democratic integrity of our societies. And in this very initial idea, we can already find a research paradox: the internal democracy of such organisations is far from optimal and, therefore, poses important problems for considering them as references of good democratic standards. 

 The text presented adheres to this perspective to lay the foundations for a research agenda aimed at improving the internal democracy of political organisations. While I share this perspective with the authors, the text submitted is often overcritical or unbalanced in highlighting the weaknesses of how internal democracy is performed.

 Aligned with the previous, some statements seem to me inadequately justified in the text. While the idea that they aim to transmit seems clear, my personal opinion is that they undermine in excess the lack of quality of internal democracy, upgrading problems that (IMHO) are not generalised to an over critical perspective where it might seem that the lack of democracy is the rule or, even, the aim of such internal processes of decision-making: 

 P.4: "Between the citizen and the state sits a dense intermediate layer of organizations that are themselves internally democratic –or at least claim to be. "

 P. 5: "Legal frameworks are almost universally inadequate" 

 I would suggest that the authors maintain the research angle and the critical perspective (I insist, I share it with them), but to tone down some statements that might make the reader get a wrong impression of how internal democracy works.  

 Secondly, the third point of the text (The digital dimension: shared vulnerabilities and organization-specific risks), merges many different elements, and I would welcome more clarity on its development: 

 1) Regarding the distinct use of voting and participation technologies: although both aim to increase the internal democratic engagement, technologies for voting are very different from technologies for social participation, but they are presented in the text as a single type of  technology

 2) One also needs to acknowledge that the standards that we use in elections (national, parliamentary,...) are very difficult to achieve at a lower level of democracy (i.e. internal organisations' democracy). The resources needed for securing electoral integrity would be quite high and would disallow the use of technology at a low level. This being said, the agenda might also focus on developing quality standards for the use of technology, setting up practices to improve the integrity of elections when using technology. 

 3) I would suggest making the distinction between technologies clearer. Voter identification, voting technologies, social media,... the text presents them as a continuum that is not logically sustained. I acknowledge the complexity of tackling such a wide topic in a few pages, and I feel the authors did a great job when preparing the text, but I still feel this distinction should be clearer. 

 My comments do not aim to undermine the interest of the text, but to provide avenues for improvement.

Where applicable, are recommendations and next steps explained clearly for others to follow? (Please consider whether others in the research community would be able to implement guidelines or recommendations and/or constructively engage in the debate)

Yes

Does the article adequately reference differing views and opinions?

Yes

Are all factual statements correct, and are statements and arguments made adequately supported by citations?

Partly

Is the rationale for the Open Letter provided in sufficient detail? (Please consider whether existing challenges in the field are outlined clearly and whether the purpose of the letter is explained)

Yes

Is the Open Letter written in accessible language? (Please consider whether all subject-specific terms, concepts and abbreviations are explained)

Yes

Reviewer Expertise:

internet voting, voting technologies, electronic democracy, digital sociology

I confirm that I have read this submission and believe that I have an appropriate level of expertise to confirm that it is of an acceptable scientific standard, however I have significant reservations, as outlined above.

# Reviewer report: Reviewer response for version 1

**DOI:** 10.21956/openreseurope.25660.r74157

**Published:** 2026-06-06

**Authors:** John Ishiyama

**Affiliations:** 1 University of North Texas, Denton, Texas, USA

This article seeks to establish a new research agenda to examine electoral integrity within political organizations. It offers a number of themes that research could explore. The paper correctly in my view identifies a significant gap in the existing electoral integrity literature and points to ways in which scholars may address this gap. The piece is well written and quite clear, and I think it makes an important contribution. I am very intrigued by some of the ideas about topics that could be explored

Where applicable, are recommendations and next steps explained clearly for others to follow? (Please consider whether others in the research community would be able to implement guidelines or recommendations and/or constructively engage in the debate)

Yes

Does the article adequately reference differing views and opinions?

Yes

Are all factual statements correct, and are statements and arguments made adequately supported by citations?

Yes

Is the rationale for the Open Letter provided in sufficient detail? (Please consider whether existing challenges in the field are outlined clearly and whether the purpose of the letter is explained)

Yes

Is the Open Letter written in accessible language? (Please consider whether all subject-specific terms, concepts and abbreviations are explained)

Yes

Reviewer Expertise:

Comparative politics, political parties

I confirm that I have read this submission and believe that I have an appropriate level of expertise to confirm that it is of an acceptable scientific standard.

# Reviewer report: Reviewer response for version 1

**DOI:** 10.21956/openreseurope.25660.r73642

**Published:** 2026-05-29

**Authors:** Yusuf Abubakar Wara

**Affiliations:** 1 Kastamonu University, Kastamonu, Kastamonu, Turkey

The open letter “Time to start a research agenda on electoral integrity within political organizations” is both timely and important. It successfully identifies a significant gap in the existing literature on electoral integrity, which has so far focused almost exclusively on national elections, while overlooking the internal electoral dynamics of the major political actors that shape those very processes. Furthermore, the letter draws attention to emerging challenges brought about by the digitalization of elections, including new risks of electoral fraud and exclusion.

 The letter’s originality also lies in its call to scrutinize and standardize the integrity of internal elections within key political organizations—such as political parties, trade unions, and civic or youth bodies. The letter claims that political organizations' internal electoral integrity is understudied despite its relevance for democratic legitimacy. It stresses that open, responsible, and credible internal elections in parties, unions, and civil society groups boost public confidence, democratic consolidation, and electoral integrity. By reminding political scientists that these organizations function as essential institutions of democracy, we deduce from the letter that when these political organizations’ internal accountability weakens, democratic consolidation suffers as a result. This observation further indicate the relevance of the prospective research.

 As a constructive suggestion for further development, the authors might consider developing a set of standardized indicators to measure the internal electoral integrity of political organizations. Measurable criteria for transparency, participation, and inclusiveness within their internal elections should be established to allow for systematic comparison.

 Furthermore, a comparative analysis between the integrity of national elections and that of internal party or organizational elections could be valuable. Such an analysis would help determine how the integrity—or lack thereof—of internal electoral processes influences the quality and credibility of national elections.

Where applicable, are recommendations and next steps explained clearly for others to follow? (Please consider whether others in the research community would be able to implement guidelines or recommendations and/or constructively engage in the debate)

Yes

Does the article adequately reference differing views and opinions?

Yes

Are all factual statements correct, and are statements and arguments made adequately supported by citations?

Yes

Is the rationale for the Open Letter provided in sufficient detail? (Please consider whether existing challenges in the field are outlined clearly and whether the purpose of the letter is explained)

Yes

Is the Open Letter written in accessible language? (Please consider whether all subject-specific terms, concepts and abbreviations are explained)

Yes

Reviewer Expertise:

Civil Society, Political Participation, Digital Politics, Democracy, and Political Parties.

I confirm that I have read this submission and believe that I have an appropriate level of expertise to confirm that it is of an acceptable scientific standard.

# Reviewer report: Reviewer response for version 1

**DOI:** 10.21956/openreseurope.25660.r74158

**Published:** 2026-05-18

**Authors:** Régis Dandoy

**Affiliations:** 1 Universidad San Francisco de Quito, Quito, Ecuador

This is a strong and ambitious open letter. The central contribution is clear: electoral integrity scholarship has largely ignored the “meso-level” of democratic organization. The open letter is conceptually coherent, empirically motivated, and normatively relevant.

 Here are my modest suggestions and remarks:

 1. The paper assumes that concepts developed for state-administered public elections (eg. electoral integrity) can travel relatively smoothly into organizational settings. This is plausible, but you need a more explicit discussion of whether organizational elections are genuinely “electoral” in the same sense; or whether “integrity” means the same thing in organizations, unions, NGOs, and parties. For example, an NGO board election may prioritize expertise or mission coherence rather than majoritarian representation. Not all democratic organizations are organized around electoral competition, and that alternative traditions of collective decision-making - including consensus, rotational leadership, cadre selection, and deliberative governance - may produce different integrity standards. You assume that competitive organizational elections are normal and that they claim democratic legitimacy, but in many parties, the internal legitimacy often depends on consultation, ideological legitimacy, or collective leadership, rather than open electoral competition.

 2. It is maybe a deliberate choice of the authors, but the bibliography is overwhelmingly Western, anglophone, European, and institutionalist. What about including the Global South party, union and NGO literature, for instance on clientelism, factionalism, informal institutions, and/or competitive authoritarianism ? As you know, in many African, Latin American, and South Asian parties, unions and NGOs the internal elections are symbolic and patronage is important.

 3. Here are two potential additions to your future research agenda:

 - Informal institutions, as much organizational power operates outside formal elections. Eg. informal veto players, clientelism, factions, external donors, etc.

 - Internal organizational elections under authoritarianism or democratic backsliding. Eg. state-regulated NGOs, regime-controlled unions, etc.

Where applicable, are recommendations and next steps explained clearly for others to follow? (Please consider whether others in the research community would be able to implement guidelines or recommendations and/or constructively engage in the debate)

Yes

Does the article adequately reference differing views and opinions?

Yes

Are all factual statements correct, and are statements and arguments made adequately supported by citations?

Yes

Is the rationale for the Open Letter provided in sufficient detail? (Please consider whether existing challenges in the field are outlined clearly and whether the purpose of the letter is explained)

Yes

Is the Open Letter written in accessible language? (Please consider whether all subject-specific terms, concepts and abbreviations are explained)

Yes

Reviewer Expertise:

Elections, Comparative politics

I confirm that I have read this submission and believe that I have an appropriate level of expertise to confirm that it is of an acceptable scientific standard.
